# Prediction of virological failure in HIV-infected individuals treated with cART in Suriname

**DOI:** 10.1186/1758-2652-13-S4-P56

**Published:** 2010-11-08

**Authors:** MP Bleijenberg, WFW Bierman, C Jaglall, MW Heymans, MC ten Doesschate, PT Nieuwkerk, PH Smits, M van Eer, S Vreden

**Affiliations:** 1 VUmc, Internal Medicine, Amsterdam, Netherlands; 2 UMCG, Internal Medicine, Groningen, Netherlands; 3 RGD (regional health service), General Practicioner, Paramaribo, Suriname; 4 VUmc, Epidemiology, Amsterdam, Netherlands; 5 UMCG, Psychiatry, Groningen, Netherlands; 6 AMC, Medical Psychology, Amsterdam, Netherlands; 7 Slotervaart Hospital, Molecular Biology, Amsterdam, Netherlands; 8 Diakonessenhuis, Internal Medicine, Paramaribo, Suriname; 9 AZP, Internal Medicine, Paramaribo, Suriname

## Purpose

In Suriname, a South American country with 500.000 inhabitants and an estimated prevalence of HIV infection of 4%, virological monitoring of combination antiretroviral therapy (cART) is uncommon. Therefore, the virological response to cART is poorly known. The objective of this study is to determine the virological response and to identify risk factors for virological failure among HIV-infected patients in Suriname. An additional aim is to develop and validate a prediction model that could be used to identify patients at risk for virological failure.

## Methods

100 HIV-infected individuals, treated with cART participated in this study. Patients were selected from 2 major hospitals and 4 general medicine practices in Paramaribo, Suriname. Patients were eligible for this study if they were prescribed cART for 6 months or more, their last drug pick-up date was no longer than 9 months before inclusion and if they were at least 18 years old. To assess risk factors multiple questionnaires were conducted considering HIV stigma (HIV Stigma scale), Social Support (based on the Norbeck Social Support Questionnaire), Depression (CES-D scale), Quality of life (based on the HIV questionnaire of the Medical Outcomes Study) and Beliefs about Medications (Beliefs about Medication Questionnaire). In addition, HIV RNA concentrations were measured in dried blood spots. A detectable viral load (HIV RNA > 300 copies/mm^3^) was considered as virological failure.

## Results

19% of all participants had a detectable viral load (range 463-319815). A significant correlation was found between virological failure and a lower level of social support (r = -0.0357, p = 0.00), younger age (r = -0.245, p = 0.014), and higher perceived personalized HIV stigma (r = 0.206, p = 0.039). A prediction model was formed consisting of three variables: age, social support and personalized HIV stigma. This model has a discriminatory power of 0.7993 (receiver operating characteristic) and an explained variance of 0.252 (R^2^).

## Conclusions

In this Surinamese cohort, 19% of patients treated with cART are demonstrating virological failure. A prediction model consisting of age, social support en personalized stigma scales can predict for almost 80% whether a viral load will be detectable or not. This prediction model could assist in identifying patients at risk for virological failure, especially in situations where virological monitoring is unavailable.

